# Transient receptor potential melastatin 3 dysfunction in post COVID-19 condition and myalgic encephalomyelitis/chronic fatigue syndrome patients

**DOI:** 10.1186/s10020-022-00528-y

**Published:** 2022-08-19

**Authors:** Etianne Martini Sasso, Katsuhiko Muraki, Natalie Eaton-Fitch, Peter Smith, Olivia Ly Lesslar, Gary Deed, Sonya Marshall-Gradisnik

**Affiliations:** 1 The National Centre for Neuroimmunology and Emerging Diseases, Menzies Health Institute Queensland, Griffith University, Gold Coast, QLD Australia; 2 Consortium Health International for Myalgic Encephalomyelitis, National Centre for Neuroimmunology and Emerging Diseases, Menzies Health Institute Queensland, Griffith University, Gold Coast, QLD Australia; 3 School of Pharmacy and Medical Science, Griffith University, Gold Coast, QLD Australia; 4 Laboratory of Cellular Pharmacology, School of Pharmacy, Aichi-Gakuin University, Nagoya, Japan; 5 Clinical Medicine, Griffith University, Gold Coast, QLD Australia; 6 LifeSpan Medicine, Los Angeles, CA USA; 7 Cingulum Health, Rosebery, NSW Australia; 8 Mediwell Medical Clinic, Coorparoo, QLD Australia

**Keywords:** Post COVID-19 condition, Coronavirus, Myalgic encephalomyelitis/chronic fatigue syndrome, Transient receptor potential melastatin 3, Natural killer cells, Whole-cell patch clamp electrophysiology, SARS-CoV-2

## Abstract

**Background:**

Myalgic encephalomyelitis/chronic fatigue syndrome (ME/CFS) is a severe multisystemic condition associated with post-infectious onset, impaired natural killer (NK) cell cytotoxicity and impaired ion channel function, namely Transient Receptor Potential Melastatin 3 (TRPM3). Long-term effects of severe acute respiratory syndrome coronavirus 2 (SARS-CoV-2) virus has resulted in neurocognitive, immunological, gastrointestinal, and cardiovascular manifestations recently recognised as post coronavirus disease 2019 (COVID-19) condition. The symptomatology of ME/CFS overlaps significantly with post COVID-19; therefore, this research aimed to investigate TRPM3 ion channel function in post COVID-19 condition patients.

**Methods:**

Whole-cell patch-clamp technique was used to measure TRPM3 ion channel activity in isolated NK cells of N = 5 ME/CFS patients, N = 5 post COVID-19 patients, and N = 5 healthy controls (HC). The TRPM3 agonist, pregnenolone sulfate (PregS) was used to activate TRPM3 function, while ononetin was used as a TRPM3 antagonist.

**Results:**

As reported in previous research, PregS-induced TRPM3 currents were significantly reduced in ME/CFS patients compared with HC (p = 0.0048). PregS-induced TRPM3 amplitude was significantly reduced in post COVID-19 condition compared with HC (p = 0.0039). Importantly, no significant difference was reported in ME/CFS patients compared with post COVID-19 condition as PregS-induced TRPM3 currents of post COVID-19 condition patients were similar of ME/CFS patients currents (p > 0.9999). Isolated NK cells from post COVID-19 condition and ME/CFS patients were resistant to ononetin and differed significantly with HC (p < 0.0001).

**Conclusion:**

The results of this investigation suggest that post COVID-19 condition patients may have impaired TRPM3 ion channel function and provide further evidence regarding the similarities between post COVID-19 condition and ME/CFS. Impaired TRPM3 channel activity in post COVID-19 condition patients suggest impaired ion mobilisation which may consequently impede cell function resulting in chronic post-infectious symptoms. Further investigation into TRPM3 function may elucidate the pathomechanism, provide a diagnostic and therapeutic target for post COVID-19 condition patients and commonalities with ME/CFS patients.

**Supplementary Information:**

The online version contains supplementary material available at 10.1186/s10020-022-00528-y.

## Background

Severe acute respiratory syndrome coronavirus 2 (SARS-CoV-2) is the infectious agent causing a highly transmissible respiratory disease called coronavirus disease 2019 (COVID-19), which in March 2020 was declared a pandemic (Cucinotta and Vanelli 2020; Pollard et al. 2020). As of April 2022, SARS-CoV-2 has infected more than 500 million people worldwide and has caused over six million deaths (World Health Organization 2022). Importantly, not all patients return to their usual state of health after the acute phase of COVID-19 as approximately 30% of these patients develop post COVID-19 condition (Logue et al. 2021; Taquet et al. 2021). Post COVID-19 condition is defined by the World Health Organization (WHO) as patients with a history of probable or confirmed SARS-CoV-2 infection, usually three months from the acute infection with symptoms that last for two or more months and that an alternative diagnosis cannot explain (World Health Organization 2021). Patients with post COVID-19 condition report persistent and prolonged symptoms including chronic fatigue, cognitive difficulties, post-exertional malaise, dyspnoea, myalgia and sleep disturbances (Mantovani et al. 2021; Komaroff and Bateman 2020; Nalbandian et al. 2021; Carfi et al. 2020; Townsend et al. 2020; Poenaru et al. 2021).

It was expected that patients with severe COVID-19 infections, who were admitted to Intensive Care Units or required invasive mechanical ventilation, would require follow-up after the acute phase of SARS-CoV-2 infection; however, the development of long-term sequelae in post COVID-19 condition does not appear to be restricted to those patients (Lledo et al. 2022). Previous investigations have demonstrated that post COVID-19 condition occurs independently of the duration and severity of COVID-19 as the authors reported that post COVID-19 condition was also found after mild or asymptomatic SARS-CoV-2 infection, hospitalised, and non-hospitalised patients (Lledo et al. 2022; Rello et al. 2021; Seessle et al. 2022). Overall, post COVID-19 condition has significantly overburdened the healthcare system, and therefore should be considered a public health emergency (Lledo et al. 2022; Rello et al. 2021).

Symptoms observed in post COVID-19 condition resemble myalgic encephalomyelitis/chronic fatigue syndrome (ME/CFS). ME/CFS is a debilitating, multifactorial, acquired condition characterised by post-exertional neuroimmune exhaustion (PENE) and symptomatology which impacts neuro-cognitive function, autonomic, endocrine, and immune systems (Carruthers et al. 2011; Perrin et al. 2020). Published reports of post COVID-19 condition described those patients as experiencing exacerbated symptoms following physical activity and stress (Poenaru et al. 2021; Lledo et al. 2022; Seessle et al. 2022; Davis et al. 2021; Balinas et al. 2021; Havervall et al. 2021), a phenomenon also seen in ME/CFS (Carruthers et al. 2011; Balinas et al. 2021). The symptomatology presented in both conditions has a profound impact on everyday functioning (disrupted work, social, and home life) and the quality of life (QoL) of patients (World Health Organization 2022; Lledo et al. 2022; Seessle et al. 2022; Balinas et al. 2021; Havervall et al. 2021; Eaton-Fitch et al. 2020). Another interesting aspect associating ME/CFS and post COVID-19 condition is the abundance of evidence reporting that onset of ME/CFS can follow viral infections, such as Epstein–Barr Virus, Q fever, influenza, and other coronaviruses (Komaroff and Bateman 2020; Poenaru et al. 2021; Hickie et al. 2006; Bansal et al. 2012). Recently, SARS-CoV-2 has received attention as a potential infectious trigger for ME/CFS; however, further research is required to confirm this hypothesis (Poenaru et al. 2021; Araja et al. 2021; Kedor et al. 2021).

The estimate is that ME/CFS affects 17 to 24 million people worldwide (Lim et al. 2020). Due to the absence of an established diagnostic test, diagnosis of ME/CFS is complex and imprecise (Westermeier et al. 2022). Currently, ME/CFS diagnosis is based on different definitions: the Fukuda or Centers for Disease Control and Prevention (CDC) criteria (1994) (Fukuda et al. 1994), Canadian Consensus Criteria (CCC, 2003) (Carruthers et al. 2003) and the International Consensus Criteria (ICC, 2011) (Carruthers et al. 2011). Although the pathophysiology is still not completely clear, previous studies have reported ME/CFS as a potential channelopathy and demonstrated the importance of Transient Receptor Potential Melastatin 3 (TRPM3) channel dysfunction in natural killer (NK) cells from ME/CFS patients (Marshall-Gradisnik et al. 2016; Nguyen et al. 2017; Cabanas et al. 2018, 2019a).

Human NK cells are large granular lymphocytes of the innate immune system abundant in peripheral blood but also widespread in peripheral tissues (Bjorkstrom and Ponzetta 2021; Vivier et al. 2008). These cells are identified according to the surface expression and density of CD56 (neural cell-adhesion molecule) and CD16 (FcγIII receptor, the low-affinity receptor for IgG), and have a protective role in several inflammatory conditions (Marshall-Gradisnik et al. 2016; Caligiuri 2008; Bryceson et al. 2006). The role of NK cells includes the production of cytokines, and cytotoxicity against tumour and/or viral-infected cells (Eaton-Fitch et al. 2019; Bjorkstrom et al. 2022). Importantly, NK cells require calcium (Ca^2+^) to regulate cellular functions including NK cell cytotoxicity (Anasetti et al. 1987; Henkart 1985; Kass and Orrenius 1999; Schwarz et al. 2013) and impaired Ca^2+^ mobilisation has been found in patients with ME/CFS (Nguyen et al. 2017).

In lymphocytes, the influx of Ca^2+^ is dependent on Ca^2+^ release activated channels (CRAC) and transient receptor potential (TRP) channels (Berridge et al. 2003). TRPM3 is a cation channel belonging to the mammalian TRP ion channel superfamily that are expressed in various cells and tissues. Specifically, TRPM3 is widely expressed in cells of the central nervous system in particular sensory ganglia, pancreatic beta islets, cardiovascular organs, skeletal muscle, genitourinary and immune cells (Montell et al. 2002; Nguyen et al. 2016). While TRPM3 is a non-selective cation channel, it is highly permeable to Ca^2+^ and contributes to biological processes including the activation of phospholipase A2, the Ca^2+^-dependent protein lipase C (PLC) and extracellular signal-regulated kinase (ERK), cell differentiation and division, apoptosis, transcriptional events, cell adhesion, immune synapse formation, granule polarisation, and release of cytolytic proteins (Schwarz et al. 2013; Clapham 2007).

Electrophysiological investigations using whole-cell patch-clamp technique reported a significant reduction in TRPM3 ion channel activity after activation with pregnenolone sulfate (PregS) and nifedipine in NK cells from ME/CFS patients (Cabanas et al. 2018, 2019a). Furthermore, ionic currents in ME/CFS patients were resistant to modulation using the TRPM3-antagonist ononetin in the presence of PregS and nifedipine (Cabanas et al. 2018, 2019a). The loss of TRPM3 ion channel activity has been validated and may have a potential role in the aetiology and pathomechanism of ME/CFS.

Despite several reports suggesting an overlap between post COVID-19 condition and ME/CFS, to the authors’ knowledge there is no laboratory-based investigation which confirms these similarities. The aim of this investigation was to determine the activity of TRPM3 ion channels using whole-cell patch-clamp measurements in isolated NK cells from post COVID-19 condition, ME/CFS and healthy control (HC) after modulation with PregS and ononetin.

## Methods

### Participant recruitment

Post COVID-19 condition patients were recruited through clinician referral and met the WHO clinical case definition of post COVID-19 condition by Delphi consensus (World Health Organization 2021). While five ME/CFS patients and five HC were recruited using the National Centre for Neuroimmunology and Emerging Diseases (NCNED) patient database between July 2021 and April 2022. Eligible participants were between 18 and 65 years of age. All ME/CFS patients had previously received a confirmed medical diagnosis and were screened using a comprehensive online questionnaire corresponding with the Fukuda (CDC) (Fukuda et al. 1994), CCC (Carruthers et al. 2003) and ICC (Carruthers et al. 2011) case definitions. All five HC reported no incidence of fatigue and were in good health without evidence of illness. Participants were excluded from this study if they were pregnant or breastfeeding or reported a previous alcohol abuse or chronic illness (for example, autoimmune diseases, cardiovascular disease, diabetes, metabolic syndrome, thyroid disease, malignancies, insomnia, chronic fatigue, and primary psychological disorders) or were obese (Body Mass Index (BMI) ≥ 30). No participants reported use of opioids or any other pain killers in the preceding 3 months as well as pharmacological agents that directly or indirectly influence TRPM3 or Ca^2+^ signalling. Participants were provided with the option to cease any conflicting medications for a minimum of 14 days prior to blood donation with the approval of their physician. All participants completed an online questionnaire to provide sociodemographic background, medical history, medications, and symptom history for post COVID-19 condition and ME/CFS. Symptoms were categorised according to the following: (i) cognitive difficulties (slowed thought, impaired concentration and short term memory loss); (ii) pain (headaches, muscle pain and multi-joint pain); (iii) sleep disturbances (unrefreshing sleep, frequent awakenings, prolonged sleep, reversed sleep cycle); (iv) cardiovascular symptoms (orthostatic intolerance, heart palpitations, light headedness and dizziness); (v) respiratory symptoms (air hunger, laboured breathing); (vi) thermostatic intolerances (subnormal body temperature, abnormal sweating episodes, hot flushes and cold extremities); (vii) sensory disturbances (sensitivity to touch, light, odour, taste, sound, movement, and poor balance or coordination); (viii) urinary disturbances (changes to urination frequency and urgency to urinate); (ix) immune disturbances (sore throat, tender lymph nodes, new allergies/sensitivities); and (x) gastrointestinal disturbances (bloating, diarrhoea or other changes in bowel movement). The 36-item short form health survey (SF-36) was used to assess patient QoL, reported data across the eight survey domains were scored on a 0 to 100 range whereby 0% = no QoL and 100% = full QoL (Stevenson 1996). The WHO Disability Assessment Schedule (DAS) was used to determine level of disability, scores were converted to a 0 to 100 percentage range whereby 0% = no disability or 100% = full disability (Andrews et al. 2009). This investigation was approved by the Griffith University Human Research Ethics Committee (GU/2019/1005) and Gold Coast University Hospital Human Research Ethics Committee (HREC/2019/QGC/56469).

### Peripheral blood mononuclear cell isolation and natural killer cell isolation

A total of 84 ml of whole blood was collected in ethylendiaminetetraacetic acid (EDTA) tubes via venepuncture by a qualified phlebotomist from each participant between 8:00 am and 12:00 pm at collection locations including Griffith University, Toowoomba Hospital, Robina Hospital, Royal Brisbane and Women’s Hospital, Sunshine Coast University Hospital, and private laboratories in South East Queensland and North East New South Wales. Routine full blood analysis was performed within four hours of collection for red blood cell count, white blood cell count and granulocyte cell count for each participant at Gold Coast University Hospital or private laboratories, Australia.

Samples were provided to the laboratory de-identified using a unique alpha-numeric code. Peripheral blood mononuclear cells (PBMCs) were isolated from 80 ml of whole blood by centrifugation over a density gradient medium (Ficoll-Paque Premium; GE Healthcare, Uppsala, Sweden) as previously described (Brenu et al. 2011; Munoz and Leff 2006). PBMCs were stained with trypan blue (Invitrogen, Carlsband, CA, USA) to determine cell count and cell viability using an automatic cell counter (TC20 Automated cell counter, Bio-Rad, Laboratories, Hercules, CA). PBMCs were adjusted to a final concentration of 5 × 10^7^ cells/ml for NK cell isolation.

NK cells were isolated by immunomagnetic selection using an EasySep Negative Human NK Cell Isolation Kit (Stem Cell Technologies, Vancouver, BC, Canada). NK cell purification was determined using flow cytometry. NK cells were incubated for 20 min at room temperature in the presence of CD56 APC (0.25 μg/20 μl) and CD3 PE Cy7 (0.25 μg/5 μl) monoclonal antibodies (Becton Dickinson (BD) Bioscience, San Jose, CA, USA) as previously described (Nguyen et al. 2017). Cells were washed and resuspended in 200 ml of stain buffer (BD Bioscience, New Jersey, USA) and acquired at 10,000 events using the Accuri C6 (BD Biosciences, San Diego, CA, USA). Using forward and side scatter, the lymphocyte population was gated while acquiring the sample. The NK cell population was then identified using phenotypic surface expression as CD3^−^CD56^+^. NK cells purity ≥ 90% was acceptable for this study as seen in Additional file 1: Fig. S1 where NK cells purity from ME/CFS, Post COVID-19 condition and HC groups is represented and no statistical difference was found between groups.

### Whole-cell electrophysiology recording

Patch-clamp technique was used to determine the respective activity of TRPM3 in NK cells from post COVID-19 condition, HC and ME/CFS patients. Electrophysiological recordings were performed with borosilicate glass capillary electrodes with an outside diameter of 1.5 mm and inside diameter of 0.86 mm (Harvard Apparatus, Holliston, MA, USA). Pipette resistance when filled with pipette solution was 8–12 MΩ. The pipettes were mounted on a CV203BU head-stage (Molecular Devices, Sunnyvale, CA, USA) connected to a 3-way coarse manipulator and a micro-manipulator (Narishige, Tokyo, Japan). Electrical signals were amplified and recorded using an Axopatch 200B amplifier and pClamp 10.7 software (Molecular Devices, Sunnyvale, CA, USA). Data were filtered at 5 kHz and sampled digitally at 10 kHz via a Digidata 1440A analogue to digital converter (Molecular Devices, Sunnyvale, CA, USA). The voltage-ramp protocol was a step from a holding potential of + 10 to − 90 mV, followed by a 0.1 s ramp to + 110 mV, before returning to + 10 mV (repeated every 10 s). The liquid junction potential between the pipette and bath solutions (− 10 mV) was corrected. A leak current component was not subtracted from the recorded currents. Electrode was filled with the intracellular pipette solution containing 30 mM CsCl, 2 mM MgCl_2_, 110 mM L-Aspartic acid, 1 mM EGTA, 10 mM HEPES, 4 mM ATP, 0.1 mM GTP, adjusted pH to 7.2 with CsOH and osmolality of 290 mOsm/L with D-mannitol. The pipette solution was filtered using a 0.22 μm membrane filter (Sigma-Aldrich, St. Louise, MO, USA), divided into aliquots and stored at − 20 °C. Bath solution contained: 130 mM NaCl, 10 mM CsCl, 1 mM MgCl_2_, 1.5 mM CaCl_2_ 2H_2_O, 10 mM HEPES, adjusted pH to 7.4 with NaOH and osmolarity 300 mOsm/L with D-glucose. All reagents were purchased from Sigma-Aldrich, except for ATP and GTP that were purchased from Sapphire Bioscience. TRPM3 ionic currents on NK cells were stimulated by adding 100 μM PregS (Tocris Bioscience, Bristol, UK) to the bath solution, whereas PregS-induced TRPM3 currents were blocked by adding 10 μM ononetin (Tocris Bioscience, Bristol, UK). All measurements were performed at room temperature. The authors reduced the possibility of chloride current involvement in TRPM3 assessment by using L-Aspartic acid in the intracellular pipette solution. Cells which have unstable currents were excluded from the analysis.

### Statistical analysis

Cytometry data was exported from Accuri C6 and analysed using SPSS v26 (IBM Corp, Version 24, Armonk, NY, USA) and GraphPad Prism v9 (GraphPad Software Inc., Version 9, La Jolla, CA, USA). Electrophysiological data were analysed using pCLAMP 10.7 software (Molecular Devices, Sunnyvale, CA, USA). Origin 2021 (OriginLab Corporation, Northampton, MA, USA), SPSS v26 and GraphPad Prism v9 (GraphPad Software Inc., Version 9, La Jolla, CA, USA) were used for statistical analysis and data presentation. Outliers were identified using the ROUT method and were removed from analysis. Visual and computed methods were used to determine normality of independent data. Specifically, histogram plots and the Shapiro–Wilk normality test was used to determine normality of independent data. Statistical comparison was performed using the independent nonparametric Kruskal–Wallis (Dunn’s multiple comparisons) test. Fisher’s exact test (applying Bonferroni method) was used to determine NK cells sensitivity to ononetin. Significance was set at p < 0.05 and the data are presented as mean ± SEM unless otherwise stated.

## Results

### Participant characteristics and blood parameters

During the study period of July 2021 to April 2022, N = 5 HC, N = 5 ME/CFS patients and N = 5 post COVID-19 condition patients participated in this study. All ME/CFS patients met CCC case definition and reported no other fatigue-related illness that may account for their symptoms. Table 1 includes demographic data of the participants. The average age of participants was 39.80 ± 14.77, 41.00 ± 9.16 and 50.80 ± 8.76 for HC, ME/CFS patients and post COVID-19 condition patients respectively. Four HC, three ME/CFS and three post COVID-19 condition participants were female. No significant differences were reported for age, gender, BMI, employment status and highest level of education between groups.

**Table 1:** Participant demographics

	HC	ME/CFS	Post COVID-19 condition	P-value
Age (years)	39.80 ± 14.77	41.00 ± 9.16	50.80 ± 8.76	0.362
Gender N (%)				
Female	4 (80.0%)	3 (60.0%)	3 (60.0%)	0.756
Male	1 (20.0%)	2 (40.0%)	2 (40.0%)	
BMI (kg/m^2^)	23.06 ± 2.80	24.00 ± 3.73	27.82 ± 1.53	0.054
Employment status				
Full time	3 (60.0%)	1 (20.0%)	4 (80.0%)	0.113
Part time	1 (20.0%)	0 (0.0%)	0 (0.0%)	
Casual	1 (20.0%)	1 (20.0%)	0 (0.0%)	
Unemployed	0 (0.0%)	0 (0.0%)	0 (0.0%)	
Illness/disability	0 (0.0%)	3 (60.0%)	1 (20.0%)	
Education				
Primary education	0 (0.0%)	0 (0.0%)	0 (0.0%)	0.364
High school	1 (20.0%)	1 (20.0%)	0 (0.0%)	
Undergraduate	2 (40.0%)	2 (40.0%)	2 (40.0%)	
Postgraduate/doctoral	2 (40.0%)	2 (40.0%)	1 (20.0%)	
Other	0 (0.0%)	0 (0.0%)	2 (40.0%)	

Data presented as mean ± SD or N (%). *HC* healthy control, *ME/CFS* myalgic encephalomyelitis/chronic fatigue syndrome, *BMI* body mass index, *N* number of participants

The SF-36 and WHO DAS surveys were used to assess QoL and disability in ME/CFS patients, post COVID-19 condition and HC. As reported in Table 2, there was a significant difference in SF-36 scores between groups for the domains physical functioning (p = 0.006), physical role (p = 0.008), pain (p = 0.008), general health (p = 0.005), social functioning (p = 0.009) and vitality (p = 0.007). Mean WHO DAS scores reported significant difference between group for domains communication and understanding (p = 0.011), mobility (p = 0.010), interpersonal relationships (p = 0.008), participation in life activities (p = 0.007) and participation in society (p = 0.011). The WHO DAS domain “participation in work/school” was not assessed due to many ME/CFS patients being unemployed. All blood parameters were within normal range according to Queensland Pathology and no significant differences were reported between groups.

**Table 2:** Participant quality of life, disability scores and serology

	HC	ME/CFS	Post COVID-19 condition	P-value
*SF-36 (%)*				
Physical functioning	100.0 ± 0.0	39.00 ± 22.75	52.0 ± 31.54	**0.006**
Physical role	100.0 ± 0.0	22.50 ± 14.39	26.25 ± 27.03	**0.008**
Pain	94.00 ± 8.94	46.50 ± 17.46	38.0 ± 18.40	**0.008**
General health	77.50 ± 11.26	26.67 ± 12.0	57.5 ± 14.25	**0.005**
Social functioning	95.00 ± 11.18	27.50 ± 24.04	22.50 ± 31.12	**0.009**
Emotional role	93.27 ± 7.00	66.67 ± 36.80	61.67 ± 48.81	0.712
Emotional wellbeing	70.00 ± 12.75	63.00 ± 17.53	62.00 ± 24.39	0.853
Vitality	66.25 ± 19.06	10.0 ± 9.48	13.75 ± 6.85	**0.007**
*WHO DAS (%)*				
Communication and understanding	5.83 ± 9.13	48.33 ± 10.87	44.17 ± 21.97	**0.011**
Mobility	2.00 ± 4.47	38.00 ± 21.09	35.00 ± 23.18	**0.010**
Self-care	0.0 ± 0.0	12.50 ± 17.12	1.25 ± 2.79	0.265
Interpersonal relationships	3.75 ± 5.59	41.25 ± 15.05	16.25 ± 12.96	**0.008**
Life activities	0.0 ± 0.0	52.65 ± 33.25	58.75 ± 32.65	**0.007**
Participation in society	3.75 ± 5.59	57.51 ± 11.19	46.87 ± 26.43	**0.011**
*Full blood count*				
White cell count (4.0–11.0 × 10^9^/L)	5.94 ± 0.72	5.16 ± 1.37	5.48 ± 1.22	0.522
Lymphocytes (1.0–4.0 × 10^9^/L)	1.87 ± 0.34	1.48 ± 0.30	1.75 ± 0.49	0.247
Neutrophils (2.0–8.0 × 10^9^/L)	3.42 ± 0.40	3.09 ± 1.08	3.20 ± 0.83	0.623
Monocytes (0.1–1.0 × 10^9^/L)	0.47 ± 0.16	0.39 ± 0.15	0.38 ± 0.12	0.432
Eosinophils (< 0.6 × 10^9^/L)	0.15 ± 0.87	0.15 ± 0.76	0.10 ± 0.36	0.606
Basophils (< 0.2 × 10^9^/L)	0.04 ± 0.02	0.03 ± 0.01	0.04 ± 0.01	0.308
Platelets (140–400 × 10^9^/L)	298.6 ± 25.83	239.60 ± 40.54	263.00 ± 50.40	0.093
Red cell count (3.8–5.2 × 10^12^/L)	4.48 ± 0.37	4.96 ± 0.47	4.67 ± 0.41	0.264
Haematocrit (0.33–0.47)	0.40 ± 0.02	0.44 ± 0.04	0.41 ± 0.03	0.102
Haemoglobin (115–160 g/L)	131.8 ± 10.89	146.4 ± 10.74	139.8 ± 12.32	0.171

Data presented as mean ± SD. Reference ranges for full blood count parameters have been included in the table. Values of p < 0.05 are bolded. *HC* healthy control, *ME/CFS* myalgic encephalomyelitis/chronic fatigue syndrome, *SF-36* 36-item short form health survey, *WHO* World Health Organization, *DAS* disability assessment schedule

All ME/CFS and post COVID-19 condition patients successfully completed a questionnaire utilising the Fukuda, CCC and ICC definitions of ME/CFS. Data detailing symptom presentation responses are detailed in Table 3. The average age of illness onset was 29.8 ± 12.48 for ME/CFS patients and 50.60 ± 8.68 for post COVID-19 condition. Average disease duration was 11.20 ± 3.56 years for ME/CFS patients and 0.56 ± 0.53 years for post COVID-19 condition. All ME/CFS patients and post COVID-19 condition patients reported cognitive difficulties and sleep disturbances. Pain was reported in all ME/CFS patients and N = 4 (80%) post COVID-19 condition patients. Sensory and Immune disturbances were reported in N = 4 (80%) ME/CFS and all post COVID-19 condition.

**Table 3:** Symptom characteristics

		ME/CFS	Post COVID-19 condition
Age of diagnosis (years [mean ± SD])		29.8 ± 12.48	50.60 ± 8.68
Disease duration (years [mean ± SD])		11.20 ± 3.56	0.56 ± 0.53
Infectious onset, N (%)		3 (60.0%)	5 (100.0%)
Cognitive difficulties	Yes	5 (100.0%)	5 (100.0%)
	No	0 (0.0%)	0 (0.0%)
Pain	Yes	5 (100.0%)	4 (80.0%)
	No	0 (0.0%)	1 (20.0%)
Sleep disturbances	Yes	5 (100.0%)	5 (100.0%)
	No	0 (0.0%)	0 (0.0%)
Sensory disturbances	Yes	4 (80.0%)	5 (100.0%)
	No	1 (20.0%)	0 (0.0%)
Immune disturbances	Yes	4 (80.0%)	5 (100.0%)
	No	1 (20.0%)	0 (0.0%)
Gastrointestinal disturbances	Yes	3 (60.0%)	4 (80.0%)
	No	2 (40.0%)	1 (20.0%)
Cardiovascular disturbances	Yes	5 (100.0%)	3 (60.0%)
	No	0 (0.0%)	2 (40.0%)
Respiratory disturbances	Yes	2 (40.0%)	4 (80.0%)
	No	3 (60.0%)	1 (20.0%)
Thermostatic instability	Yes	1 (20.0%)	2 (40.0%)
	No	4 (80.0%)	3 (60.0%)
Urinary disturbances	Yes	1 (20.0%)	1 (20.0%)
	No	4 (80.0%)	4 (80.0%)

Data presented as mean ± SD and N (%). *HC* healthy control, *ME/CFS* myalgic encephalomyelitis/chronic fatigue syndrome, *N* number of participants

### TRPM3 ion channel activity after PregS stimulation in NK cells from post COVID-19 condition patients compared with HC and ME/CFS patients

**Fig. 1:**
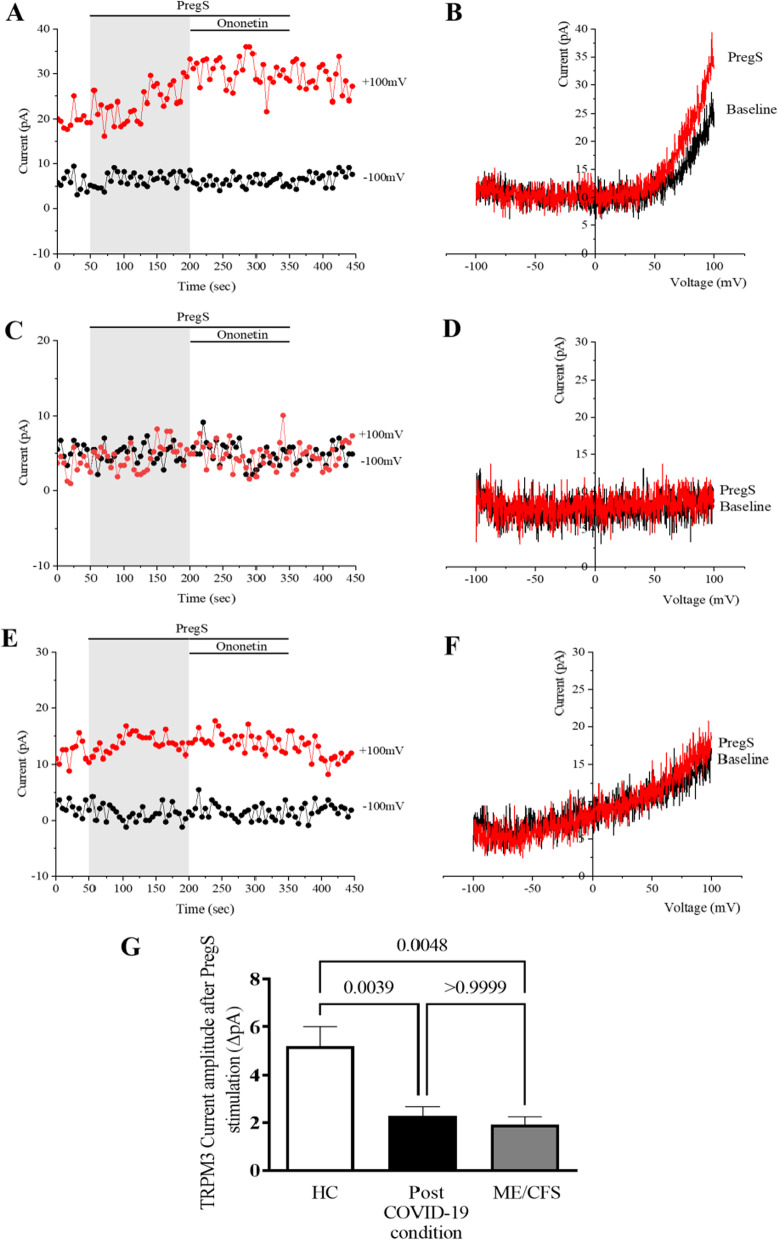
TRPM3 activity after PregS stimulation. Data were obtained under whole-cell patch-clamp conditions. Comparing all groups, amplitude of ionic current after PregS stimulation we found a significant difference (p = 0.0010). **A** A representative time-series of current amplitude at + 100 mV and − 100 mV showing the effect of 100 μΜ PregS on ionic currents in isolated NK cells from HC. **B** I–V before and after PregS stimulation in a cell corresponding with (**A**). **C** A representative time-series of current amplitude at + 100 mV and − 100 mV showing the effect of 100 μΜ PregS on ionic currents in isolated NK cells from ME/CFS patients. **D**. I–V before and after PregS stimulation in a cell as shown in (**C**). **E** A representative time-series of current amplitude at + 100 mV and − 100 mV showing the effect of 100 μΜ PregS on ionic currents in isolated NK cells from post COVID-19 condition patient. **F** I–V before and after PregS stimulation in a cell corresponding with (**E**). **G** Bar graphs representing TRPM3 current amplitude at + 100 mV after stimulation with 100 μΜ PregS in HC patients (N = 5; n = 34) compared with post COVID-19 condition patients (N = 5; n = 38) and ME/CFS patients (N = 5; n = 26). TRPM3 currents were determined as a change in amplitude from baseline to PregS induced peak as represented in time-series graphs. I–V curves were used to identify an outward rectification typical of TRPM3. N refers to number of participants and n to number of records analysed. Data are represented as mean ± SEM. *HC* healthy control, *ME/CFS* myalgic encephalomyelitis/chronic fatigue syndrome

To record TRPM3 ion channel activity in isolated NK cells from HC, ME/CFS and post COVID-19 condition patients whole-cell patch-clamp technique was used. Application of 100 μM PregS activates rapidly and reversibly endogenous TRPM3 ion channel function. Comparing all groups amplitude of ionic current after PregS stimulation we found a significant difference (p = 0.0010). As previously reported and expected, stimulation with PregS enabled measurement of a small outwardly rectifying current under voltage-clamp conditions, as well as observation of a typical shape of the TRPM3 current–voltage relationship (I–V) (Fig. 1). Following PregS application, a small ionic current was measured with a typical TRPM3-like outward rectification in NK cells isolated from HC (Fig. 1A, B). In contrast, and as previously reported (Cabanas et al. 2018, 2019a, 2019b, 2021), the amplitude of ionic current was significantly smaller (Fig. 1C, D) in NK cells from ME/CFS than HC patients after addition of PregS (Fig. 1G, p = 0.0048). Interestingly, PregS stimulation in NK cells from post COVID-19 condition patients (Fig. 1E, F) mimicked the PregS-induced result in NK cells from ME/CFS patients (Fig. 1G, p > 0.999), suggesting that NK cells from post COVID-19 condition patients also have impaired TRPM3 channel activity. In line with this, post COVID-19 condition amplitude after PregS stimulation in comparison with HC showed a significant difference (Fig. 1G, p = 0.0039).

### Modulation of PregS- evoked currents with ononetin in NK cells from post COVID-19 condition patients compared with HC and ME/CFS patients

**Fig. 2:**
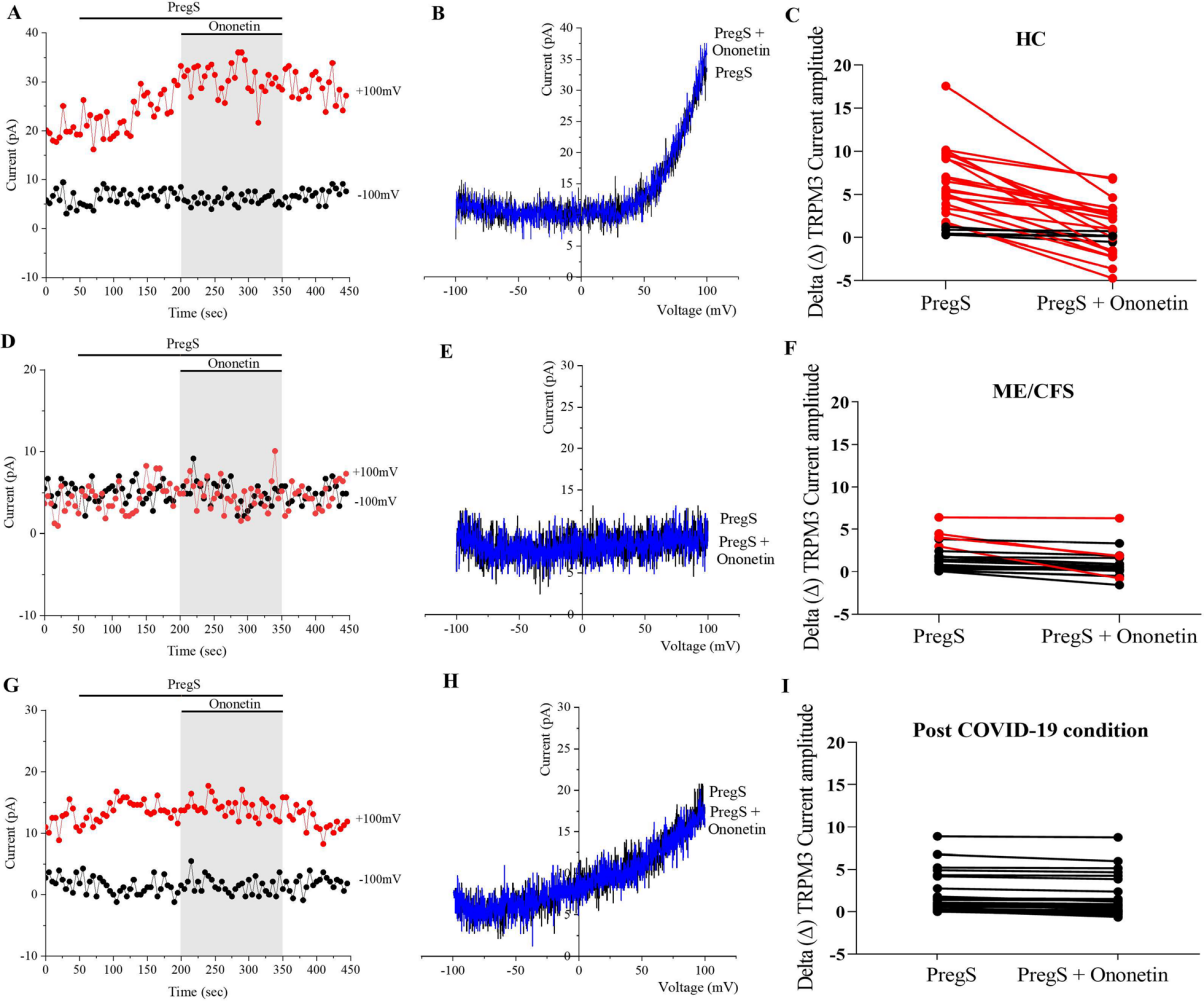
TRPM3 activity after ononetin modulation. Data were obtained under whole-cell patch-clamp conditions. **A** A representative time-series of current amplitude at + 100 mV and − 100 mV showing the effect of 10 μΜ ononetin on ionic currents in the presence of PregS in isolated NK cells from HC. **B** I–V before and after application of ononetin in a cell as shown in (**A**). **C** Scatter plots representing change of each current amplitude before and after application of ononetin in presence of PregS in all NK cells from HC. **D** A representative time-series of current amplitude at + 100 mV and − 100 mV showing the effect of 10 μΜ ononetin on ionic currents in the presence of PregS in isolated NK cells ME/CFS patients. **E** I–V before and after application of ononetin in a cell as shown in (**D**). **F** Scatter plots representing change of each current amplitude before and after application of ononetin in presence of PregS in all NK cells from ME/CFS. **G** A representative time-series of current amplitude at + 100 mV and − 100 mV showing the effect of 10 μΜ ononetin on ionic currents in the presence of PregS in isolated NK cells from post COVID-19 condition. **H** I–V before and after application of ononetin in a cell as shown in (**G**). **I** Scatter plots representing change of each current amplitude before and after application of ononetin in presence of PregS in all NK cells from post COVID-19 condition. Each cell represented as red lines indicate cells sensitive to ononetin as a reduction in amplitude was recorded. HC (N = 5; n = 29), post COVID-19 condition (N = 5; n = 27), and ME/CFS (N = 5; n = 23). N to number of participants and n to number of records analysed. *HC* healthy controls, *ME/CFS* myalgic encephalomyelitis/chronic fatigue syndrome

**Fig. 3:**
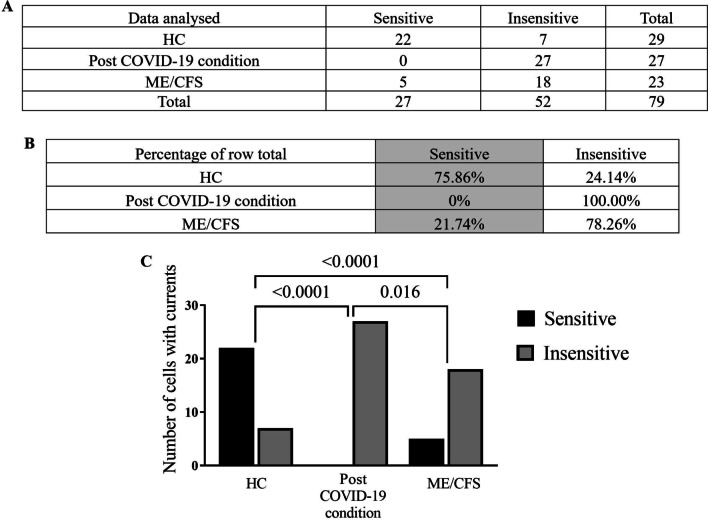
Summary TRPM3 activity after ononetin modulation. Data were obtained under whole-cell patch-clamp conditions. Table summarizing data for sensitive and insensitive cells to 10 μΜ ononetin, (**A**) absolute number and (**B**) percentage. **C** Bar graphs representing sensitive and insensitive cells to 10 μΜ ononetin, HC patients (N = 5; n = 29) compared with post COVID-19 condition patients (N = 5; n = 27) and ME/CFS patients (N = 5; n = 23). Data are analysed using Fisher’s exact test. N refers to number of participants and n to number of records analysed. *HC* healthy controls, *ME/CFS* myalgic encephalomyelitis/chronic fatigue syndrome

Ononetin effectively inhibits PregS-induced Ca^2+^-influx and ionic currents through TRPM3 ion channels (Straub et al. 2013). In this study, ononetin was used to confirm that TRPM3 activity is involved in ionic currents evoked by PregS in NK cells, 10 μM ononetin was used to modulate TRPM3 ion channel activity after PregS stimulation (Figs. 2 and 3). As previously described (Cabanas et al. 2018, 2019a, 2019b, 2021), simultaneous application of ononetin was effective to inhibit ionic currents evoked by application of PregS in isolated NK cells from HC patients (Fig. 2A-C). In contrast, and as previously shown, in NK cells from ME/CFS patients, ionic currents in the presence of PregS were mostly resistant to ononetin (Fig. 2D-F), in comparison with HC patients (Fig. 3C, p < 0.0001), indicating significant loss of the TRPM3 ion channel function in ME/CFS patients. As noted above regarding PregS data, isolated NK cells from post COVID-19 condition also showed significant difference in comparison with HC (Fig. 3C, p < 0.0001). Interestingly, the number of sensitive currents of NK cells from post COVID-19 condition were significantly lower compared with ME/CFS patients (Fig. 2G-I, Fig. 3C , p = 0.016). In Fig. 2C, F, I, scatter plots represent change of each current amplitude before and after modulation with ononetin in presence of PregS.

## Discussion

The present study provides significant and novel finding of impaired TRPM3 ion channel activity in post COVID-19 condition. To characterise TRPM3 ion channel activity, the gold standard patch-clamp technique was used to assess ion channel properties. For the first time, we report a significant reduction in amplitude of TRPM3 ion channel current after PregS stimulation in isolated NK cells from post COVID-19 condition patients compared with HC. The loss of TRPM3 ion channel activity in ME/CFS patients compared with HC is consistent with previous investigations (Cabanas et al. 2018, 2019a, 2019b, 2021). Importantly, there is no significant difference in TRPM3 ion channel activity between ME/CFS patients and post COVID-19 condition patients. Moreover, we provide evidence that ionic currents in NK cells from ME/CFS and post COVID-19 condition patients were resistant to ononetin in the presence of PregS. Although numerous studies around the world have described similarities of persistent symptoms of post COVID-19 condition and ME/CFS patients (Mantovani et al. 2021; Gonzalez-Hermosillo et al. 2021; Wong and Weitzer 2021; Sukocheva et al. 2021; Malkova et al. 2021; Yong and Liu 2022), this is the first study to report that post COVID-19 condition has an impaired function already widely proven in isolated NK cells from ME/CFS patients. Therefore, TRPM3 ion channel dysfunction in NK cells may play a pivotal role in the pathomechanism of ME/CFS and also in post COVID-19 condition.

NK cells contribute significantly to the maintenance of immune system function, which is vital to eliminate pathogen-infected cells and tumour cells (Brenu et al. 2013). Data has proposed that immune homeostasis disruption occurs in COVID-19 disease. The immunopathology of SARS-CoV-2 infection is based on dysregulation of the innate and cell-mediated immune responses (Eeden et al. 2020; Wang et al. 2020; Zheng et al. 2020). NK cell counts were remarkably lower in severe COVID-19 cases than in mild COVID-19 cases (Wang et al. 2020; Zheng et al. 2020). According to Wang and colleagues, the lymphopenia found in COVID-19 patients indicated impairment of the immune system during infection and were associated with the clinical characteristics of COVID-19 (Wang et al. 2020). Similarly, Zheng et al. suggested that the functional exhaustion of cytotoxic lymphocytes found in COVID-19 patients is associated with increased expression of the NK inhibitory receptor NKG2A. In contrast, the total number of NK and CD8 + T lymphocytes were restored and the percentage of NKG2A^+^ NK cells decreased in recovering COVID-19 patients following antiviral therapy (Zheng et al. 2020). Wilk et al. investigated peripheral immunity in severe COVID-19 using single-cell transcriptomics to characterize peripheral immune responses. In support of the above claims, these authors described that NK cells were associated with three exhaustion markers (LAG3, PDCD1 and HAVCR2) in most patients with COVID-19 (Wilk et al. 2020).

Although preliminary studies suggest the impact of NK cells reduction during SARS-CoV-2 infection, to date, neither study reported the role of these markers in the post COVID-19 condition. By contrast, the immune system dysfunction and abnormalities in NK cell functions have been the most consistent immunological features of ME/CFS, including a significant reduction in NK cells quantity and cytotoxic activity (Brenu et al. 2011, 2013; Whiteside and Friberg 1998; Hardcastle et al. 2015). The recent finding of impaired TRPM3 ion channel and Ca^2+^ mobilisation in NK cells (Marshall-Gradisnik et al. 2016; Nguyen et al. 2017, 2016; Cabanas et al. 2018, 2019a), provides further evidence of immune dysfunction in ME/CFS (Bansal et al. 2012; Eaton-Fitch et al. 2019; Lorusso et al. 2009). TRPM3 has a substantial regulatory function in Ca^2+^ signalling that is crucial for cell functions, intracellular signalling pathways, and maintaining cellular homeostasis (Schwarz et al. 2013; Nguyen et al. 2016; Clapham 2007). Dysfunction of TRPM3 in ME/CFS patients has been shown to affect Ca^2+^signalling, which has an impact on NK cells regulatory machinery and functions (Cabanas et al. 2018, 2019b). Specifically, TRPM3 on NK cells may contribute to the Ca^2+^-dependent phosphorylation of signalling proteins including phosphatidylinositol 4,5-bisphosphate 3-kinase (PI3K) and mitogen-activated protein kinases (MAPK) which result in cytokine production and cytotoxic function (Thiel et al. 2017). Therefore, impaired TRPM3 ion channels may contribute to post COVID-19 condition through consequences of impaired Ca^2+^ signalling, thus impeding Ca^2+^-dependent cellular pathways resulting in impaired NK cell cytotoxicity.

TRP channels play a role in inflammation, pain, and fever, in addition to symptoms associated with ME/CFS such as respiratory, cardiovascular, gastrointestinal, and neurocognition manifestations. Interestingly, TRP channels are expressed in many cells considered to be related to some symptoms and consequences of SARS-CoV-2 infection, thus provide potential targets for treatment and prevention of COVID-19 (Berlansky et al. 2022; Jaffal and Abbas 2021). Given that TRP ion channels are vulnerable to threats/stressors, such as viruses, further research is required to differentiate TRPM3 dysfunction in ME/CFS patients compared with post COVID-19 condition. This current investigation reported a significant difference in TRPM3 currents following ononetin modulation between post COVID-19 condition and ME/CFS patients. All NK cells analysed from post COVID-19 condition patients (N = 5, n = 27) were insensitive to ononetin, compared with 78.26% of NK cells from ME/CFS and 24.14% of NK cells from HC. This finding may suggest that the mechanisms resulting in TRPM3 ion channel dysfunction in post COVID-19 condition differs from ME/CFS patients. Further studies should be performed to identify possible causes of this TRPM3 dysfunction.

Due to the overlap observed for PregS-induced currents, TRPM3 ion channel dysfunction may provide a target for research into the pathomechanism and treatment of not only ME/CFS, but also post COVID-19 condition. In support of this, an investigation by Cabanas et al. reported that in vitro treatment of NK cells from ME/CFS patients using naltrexone hydrochloride (NTX) restored TRPM3 ion channel function and re-established Ca^2+^ homeostasis (Cabanas et al. 2019b). In a subsequent study Cabanas et al. found that ME/CFS patients taking low dose NTX (LDN) (3.0–5.0 mg/day) had restored, or normalised, TRPM3 ion channel activity compared with HC (Cabanas et al. 2021). The restoration of impaired TRPM3 ion channels consequently rebalanced the different Ca^2+^-dependent mechanisms, such as restoration of the integrity and stability of these NK cells specific signalling systems (Cabanas et al. 2019b). Therefore, future research might assess in vitro effects of NTX treatment in the restoration of physiological TRPM3 in NK cells and also the potential pharmacological benefit of LDN in post COVID-19 condition patients, as previously indicated in ME/CFS patients.

Importantly, the COVID-19 pandemic has contributed to increasing the rate of ME/CFS cases (Araja et al. 2021). Notably, future investigations into the similarity of impaired functions in NK cells from post COVID-19 condition and ME/CFS would provide benefits for patients suffering post COVID-19 condition due to existing knowledge on ME/CFS. Additionally, many ME/CFS patients face difficulties seeking diagnosis and treatment as many physicians lack awareness and understanding of ME/CFS (Kedor et al. 2021; Pheby et al. 2021). Indeed, the increase of public interest in post COVID-19 condition, along with the increase of research efforts, may contribute to additional research and development of therapeutic strategies in ME/CFS. The similarities with post COVID-19 condition represent an unprecedented opportunity to study the pathophysiology of ME/CFS (Kedor et al. 2021; Pheby et al. 2021; Nath 2020; Friedman et al. 2021).

Further investigations with a larger cohort are required to confirm the results of this pilot study. Additionally, a controlled cohort consisting of participants who have recovered from COVID-19 is suggested.

## Conclusion

We report, for the first time, TRPM3 ion channel dysfunction in post COVID-19 condition. Results of this current manuscript supports the hypothesis that there exists a pathomechanism overlap between ME/CFS patients and post COVID-19 condition. Further, SARS-CoV-2 infection may result in impaired TRPM3 ion channel dysfunction and provides a potential trigger for ME/CFS. Importantly, future investigations may examine the TRPM3 ion channels as a potential target for treatment of post COVID-19 condition.

## Supplementary Information

**Additional file 1: Fig. S1.** Natural Killer Cell Purity. NK cells purity (CD3^−^CD56^+^) was 93.90% ± 1.054 for post COVID-19 condition, 93.22% ± 3.098 for HC and 93.64% ± 1.521 for ME/CFS patients as determined by flow cytometry. NK cells were incubated for 20 min at room temperature in the presence of CD56 APC (0.25 μg/20 μl) and CD3 PE Cy7 (0.25 μg/5 μl) monoclonal antibodies (BD Bioscience, San Jose, CA, USA). Cells were acquired at 10,000 events using the Accuri C6 flow cytometer (BD Biosciences, San Diego, CA, USA). Gating strategy is as follows: (**A**) lymphocytes were gated based of SSC and FSC. (**B**) CD3 negative population was gated from selected lymphocyte population. Gating was determined using isotype controls. (**C**) NK cell purity was determined based on CD56 positive cells using the CD3 negative population. (**D**) Bar graphs representing NK cell purity (%) determined using flow cytometry methods. HC NK cell purity was 93.22% ± 3.098, post COVID-19 condition NK cell purity was 93.90% ± 1.054 and ME/CFS NK cell purity was 93.64% ± 1.521. Data presented as mean ± SEM. Abbreviation: NK, natural killer; HC, healthy controls; ME/CFS, Myalgic encephalomyelitis/chronic fatigue syndrome.

## Data Availability

The datasets generated and/or analysed during the current study are not publicly available due to confidentiality agreements but are available from the corresponding author on reasonable request.

## Abbreviations

BD: Becton Dickinson
BMI: Body mass index
Ca^2+^: Calcium
CCC: Canadian Consensus Criteria
CDC: Centers for Disease Control and Prevention
COVID-19: Coronavirus disease 2019
CRAC: Calcium release activated channels
DAS: Disability assessment schedule
EDTA: Ethylendiaminetetraacetic acid
ERK: Extracellular signal-regulated kinase
ICC: International Consensus Criteria
I–V: Current–voltage relationship
HC: Healthy controls
LDN: Low dose naltrexone
MAPK: Mitogen-activated protein kinases
ME/CFS: Myalgic encephalomyelitis/chronic fatigue syndrome
NCNED: National Centre for Neuroimmunology and Emerging Diseases
NK: Natural killer
NTX: Naltrexone hydrochloride
PBMCs: Peripheral blood mononuclear cells
PENE: Post-exertional neuroimmune exhaustion
PI3K: Phosphatidylinositol 4,5-bisphosphate 3-kinase
PLC: Protein lipase C
PregS: Pregnenolone sulfate
QoL: Quality of life
SARS-CoV-2: Severe acute respiratory syndrome coronavirus 2
SF-36: 36-Item Short Form Health Survey
SPSS: Statistical Package for the Social Sciences
TRP: Transient receptor potential
TRPM3: Transient receptor potential melastatin 3
WHO: World Health Organization
