# Supplementary figures and images for: Transient receptor potential melastatin 3 dysfunction in post COVID-19 condition and myalgic encephalomyelitis/chronic fatigue syndrome patients
Source: Mol Med. 2022 Aug 19;28:98. doi: 10.1186/s10020-022-00528-y (PMC9388968; doi:10.1186/s10020-022-00528-y)

**A**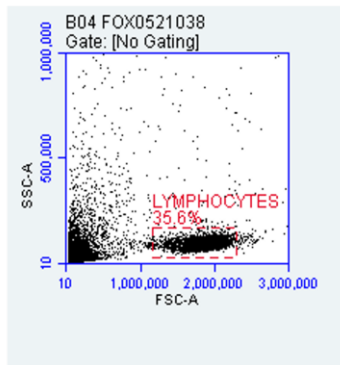**B**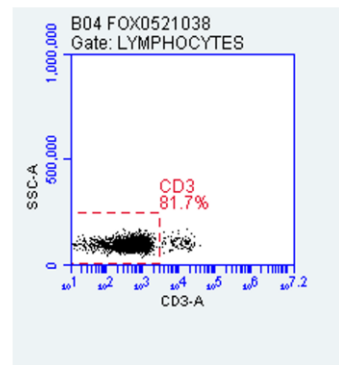**C**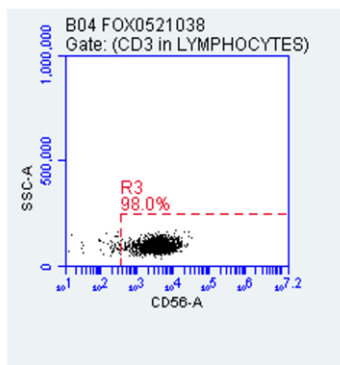**D**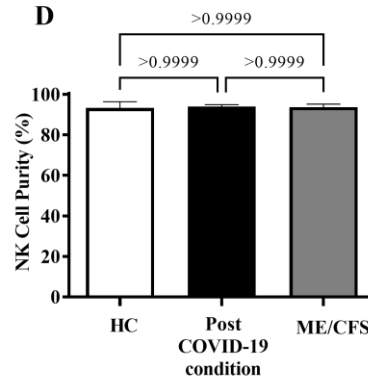

Supplement: Supplementary file 1 — Additional file 1: Fig. S1. Natural Killer Cell Purity. NK cells purity (CD3−CD56+) was 93.90% ± 1.054 for post COVID-19 condition, 93.22% ± 3.098 for HC and 93.64% ± 1.521 for ME/CFS patients as determined by flow cytometry. NK cells were incubated for 20 min at room temperature in the presence of CD56 APC (0.25 μg/20 μl) and CD3 PE Cy7 (0.25 μg/5 μl) monoclonal antibodies (BD Bioscience, San Jose, CA, USA). Cells were acquired at 10,000 events using the Accuri C6 flow cytometer (BD Biosciences, San Diego, CA, USA). Gating strategy is as follows: (A) lymphocytes were gated based of SSC and FSC. (B) CD3 negative population was gated from selected lymphocyte population. Gating was determined using isotype controls. (C) NK cell purity was determined based on CD56 positive cells using the CD3 negative population. (D) Bar graphs representing NK cell purity (%) determined using flow cytometry methods. HC NK cell purity was 93.22% ± 3.098, post COVID-19 condition NK cell purity was 93.90% ± 1.054 and ME/CFS NK cell purity was 93.64% ± 1.521. Data presented as mean ± SEM. Abbreviation: NK, natural killer; HC, healthy controls; ME/CFS, Myalgic encephalomyelitis/chronic fatigue syndrome. [file 10020_2022_528_MOESM1_ESM.pdf]
